# Multicenter Prospective Analysis of Hypertrophic Olivary Degeneration Following Infratentorial Stroke (HOD-IS): Evaluation of Disease Epidemiology, Clinical Presentation, and MR-Imaging Aspects

**DOI:** 10.3389/fneur.2021.675123

**Published:** 2021-07-16

**Authors:** Martin A. Schaller-Paule, Eike Steidl, Manoj Shrestha, Ralf Deichmann, Helmuth Steinmetz, Alexander Seiler, Sriramya Lapa, Thorsten Steiner, Sven Thonke, Stefan Weidauer, Juergen Konczalla, Elke Hattingen, Christian Foerch

**Affiliations:** ^1^Department of Neurology, University Hospital Frankfurt, Goethe-University, Frankfurt, Germany; ^2^Institute of Neuroradiology, University Hospital Frankfurt, Goethe-University, Frankfurt, Germany; ^3^Brain Imaging Center (BIC), Goethe-University, Frankfurt, Germany; ^4^Department of Neurology, Klinikum Frankfurt Höchst, Teaching Hospital of the Goethe-University, Frankfurt, Germany; ^5^Department of Neurology, Heidelberg University Hospital, Heidelberg, Germany; ^6^Department of Neurology, Klinikum Hanau, Teaching Hospital of the Goethe-University, Frankfurt, Germany; ^7^Department of Neurology, Sankt Katharinen Hospital, Teaching Hospital of the Goethe-University Frankfurt, Frankfurt, Germany; ^8^Department of Neurosurgery, University Hospital Frankfurt, Goethe-University, Frankfurt, Germany

**Keywords:** neurodegeneration, cerebellum, palatal tremor, brainstem, connectivity, tractography, Holmes tremor

## Abstract

**Introduction:** Ischemic and hemorrhagic strokes in the brainstem and cerebellum with injury to the functional loop of the Guillain-Mollaret triangle (GMT) can trigger a series of events that result in secondary trans-synaptic neurodegeneration of the inferior olivary nucleus. In an unknown percentage of patients, this leads to a condition called hypertrophic olivary degeneration (HOD). Characteristic clinical symptoms of HOD progress slowly over months and consist of a rhythmic palatal tremor, vertical pendular nystagmus, and Holmes tremor of the upper limbs. Diffusion Tensor Imaging (DTI) with tractography is a promising method to identify functional pathway lesions along the cerebello-thalamo-cortical connectivity and to generate a deeper understanding of the HOD pathophysiology. The incidence of HOD development following stroke and the timeline of clinical symptoms have not yet been determined in prospective studies—a prerequisite for the surveillance of patients at risk.

**Methods and Analysis:** Patients with ischemic and hemorrhagic strokes in the brainstem and cerebellum with a topo-anatomical relation to the GMT are recruited within certified stroke units of the Interdisciplinary Neurovascular Network of the Rhine-Main. Matching lesions are identified using a predefined MRI template. Eligible patients are prospectively followed up and present at 4 and 8 months after the index event. During study visits, a clinical neurological examination and brain MRI, including high-resolution T2-, proton-density-weighted imaging, and DTI tractography, are performed. Fiberoptic endoscopic evaluation of swallowing is optional if palatal tremor is encountered.

**Study Outcomes:** The primary endpoint of this prospective clinical multicenter study is to determine the frequency of radiological HOD development in patients with a posterior fossa stroke affecting the GMT at 8 months after the index event. Secondary endpoints are identification of (1) the timeline and relevance of clinical symptoms, (2) lesion localizations more prone to HOD occurrence, and (3) the best MR-imaging regimen for HOD identification. Additionally, (4) DTI tractography data are used to analyze individual pathway lesions. The aim is to contribute to the epidemiological and pathophysiological understanding of HOD and hereby facilitate future research on therapeutic and prophylactic measures.

**Clinical Trial Registration:** HOD-IS is a registered trial at https://www.drks.de/drks_web/navigate.do?navigationId=trial.HTML&TRIAL_ID=DRKS00020549.

## Introduction

**Figure 1:**
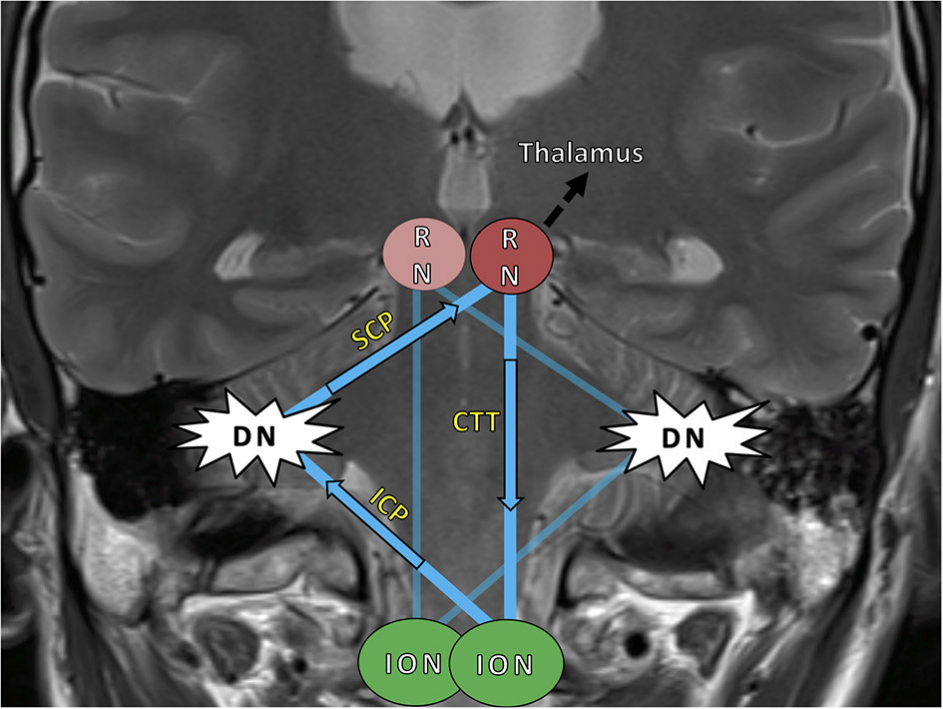
The Guillain–Mollaret triangle (GMT) (blue) and its unidirectional course of inhibitory fibers, which ascend from the dentate nucleus (DN) to the contralateral red nucleus (RN) and then descend ipsilaterally to the inferior olivary nucleus (ION). The bilateral overlapping Guillain–Mollaret triangles together form a “tilted star of David configuration” (5). Fibers of the GMT, which is also called the dentato–rubro–olivary-pathway, overlap with dentato–thalamo–cortical pathways proceeding into both hemispheres (black arrow) (10). HOD, hypertrophic olivary degeneration; SCP, superior cerebellar peduncle; ICP, inferior cerebellar peduncle; RN, red nucleus; CTT, central tegmental tract; DN, dentate nucleus; ION, inferior olivary nucleus.

Stroke is characterized by the sudden onset of a focal neurological deficit with complete or incomplete recovery over time. Some patients with infratentorial strokes, however, develop a novel clinical worsening months after the index event and with considerable impairment to their daily routine. The occurring syndrome consists of a palatal tremor (PT), vertical pendular nystagmus, and (dentatorubral) Holmes tremor of the upper limbs. This is attributed to a phenomenon called hypertrophic olivary degeneration (HOD) in which lesions to the anatomical-functional brainstem loop of the Guillain-Mollaret triangle (GMT) lead to a spatially distant degeneration of the inferior olivary nucleus (1, 2). The GMT has a unidirectional orientation of inhibitory fibers and is composed of three anatomic structures: the red nucleus (RN) in the mesencephalon, the ipsilateral inferior olivary nucleus (ION) in the medulla, and the contralateral dentate nucleus (DN) of the cerebellum (Figure 1) (3–6). It is also referred to as the dentato-rubro-olivary pathway (DROP) (7). The radiological MRI diagnosis of HOD is based on the finding of a circumscribed ION hyperintensity on T2-weighted sequences within 1 to 12 months after the index event—additional olivary hypertrophy usually after 12 months is optional (4, 8, 9).

**Figure 2:**
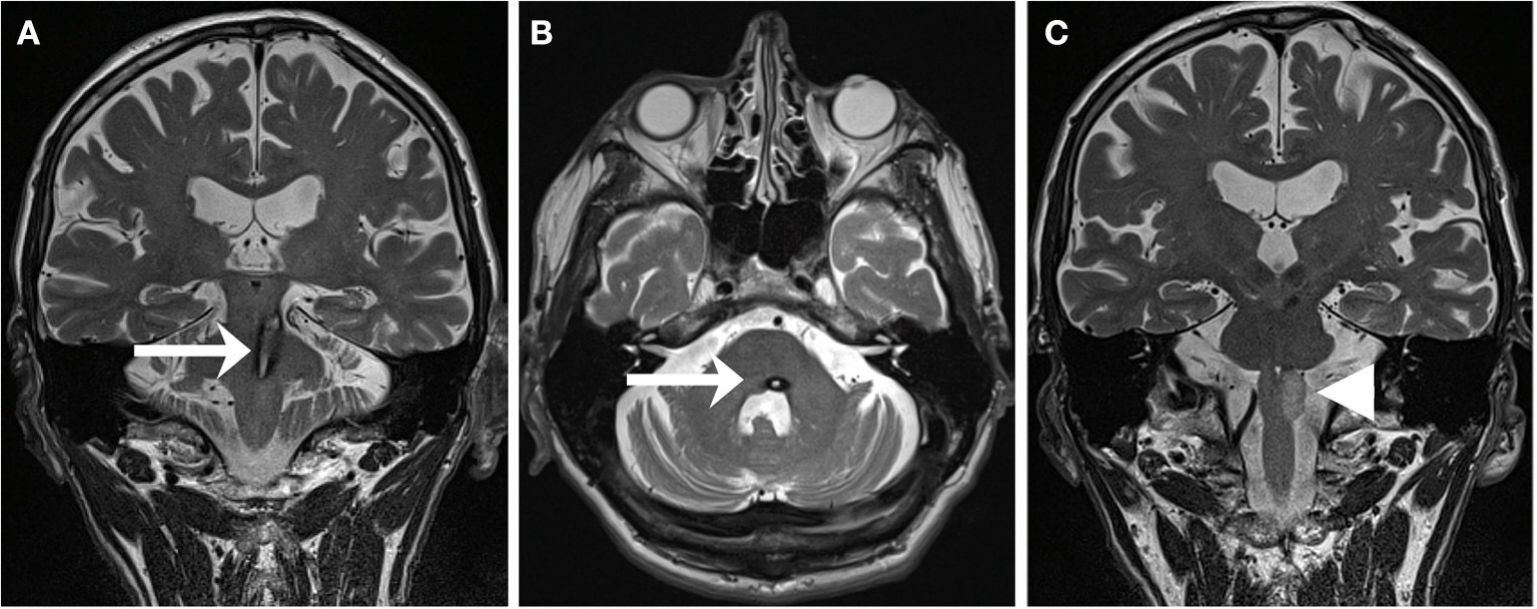
T2-weighted MRI of a 59-year-old patient suffering from pontine-mesencephalic bleeding **(A,B)** affecting the central tegmental tract on the left (arrows). Within 13 months, the patient developed a HOD **(C)** with hyperintensity of the left olive (arrowhead) accompanied by the clinical syndrome of a palatal tremor, a pendular nystagmus, and a Holmes tremor [adapted with permission from Foerch et al. (10)].

Regularly, strategic brain infarcts and intracerebral hemorrhages in the posterior fossa set lesion to the GMT and initiate a cascade of trans-synaptic events, which ultimately results in HOD (10) (Figure 2). Disinhibition of the ION leads to cerebellar malfunction and the establishment of clinical symptoms in the fine motor and pharyngeal system. Though HOD has been known for over a century, epidemiological knowledge has hardly progressed since, and neither larger clinical analyses nor prospective data are available (11, 12). A causal therapy of HOD development does not exist, and there is very limited evidence for symptomatic medical options (10, 13).

A single-center retrospective evaluation of patients with ischemic stroke and intracerebral hemorrhage has been performed previously as a proof-of-concept study. It revealed 12 patients with radiological HOD between 2010 and 2017 alone, and the majority of patients exhibited HOD symptoms (10). Based on the findings, a disease model with two overlaying GMT in a “tilted star-of-David”-configuration was established, allowing for the prediction of the side of HOD according to lesion location (Figure 1). Follow-up analyses of neuro-oncological and neuro-surgical patients revealed a frequency of HOD-occurrence after posterior fossa surgery of up to 20% depending on the surgical approach and DN injury (3, 13). Although characteristic symptoms of HOD occurred, they were missed or misinterpreted by the clinicians, and the respective radiological signs of HOD remained mostly undiscussed by radiologists (13). This confirmed that HOD diagnosis is not always recognized and may be wrongly mistaken for a novel second stroke or other pathology, such as metastasis (10, 13). However, increased awareness of HOD is pre-conditional for improved disease management. Knowledge of the disease incidence and pathophysiological mechanisms is a prerequisite for clinical surveillance of patients at risk. To follow up those patients and offer symptom-specific support, it is first required that we create a timeline of radiological HOD occurrence and the chronological onset of clinical symptoms. Concomitantly, matching fiber tract pathology to a corresponding clinical course of HOD would be of scientific value.

Advanced imaging techniques have been sporadically applied in HOD patients in this regard to visualize fiber tracts associated with HOD development. Diffusion tensor imaging (DTI) with tractography can be used to analyze the change in fiber tract volume following injury in the GMT (14–16). The use of this method systematically to investigate the HOD development and more precisely understand the injury to brainstem connectivity is promising beyond merely pinpointing down a lesion to an anatomic localization on MRI or in *ex-vivo* pathological studies. Furthermore, the distinction between affected and unaffected patients allows us to identify contributing factors to HOD occurrence. To our knowledge, no such prospective acquisition of DTI fiber tracking data in HOD patients has been described in the literature so far.

Moreover, proton density (PD)-weighted imaging was shown to be well-suited for detecting infratentorial lesions in the posterior fossa, yielding good contrast between cerebrospinal fluid (CSF) and brainstem lesions (17, 18). Transferring this to HOD imaging, it can be hypothesized that double-echo sequences (including T2- and PD-weighted datasets) are superior in the detection of HOD compared to conventional T2-weighted imaging and provide an improved detectability of the underlying lesion to the GMT.

### Objective

This study is designed to prospectively determine the frequency of HOD following ischemic or hemorrhagic lesions in the Guillain-Mollaret triangle and to examine the development of the associated clinical syndrome. As a secondary goal, the implementation of advanced imaging methods, comprising DTI for fiber tractography, will be prospectively applied to correlate the clinical findings over time with the respective fiber tract injury and generate a pathophysiological timeline of HOD development. Furthermore, the patient cohort is well-suited to compare the detectability of radiological HOD in T2- and PD-weighted data.

## Methods and Analysis

### Design

**Figure 3:**
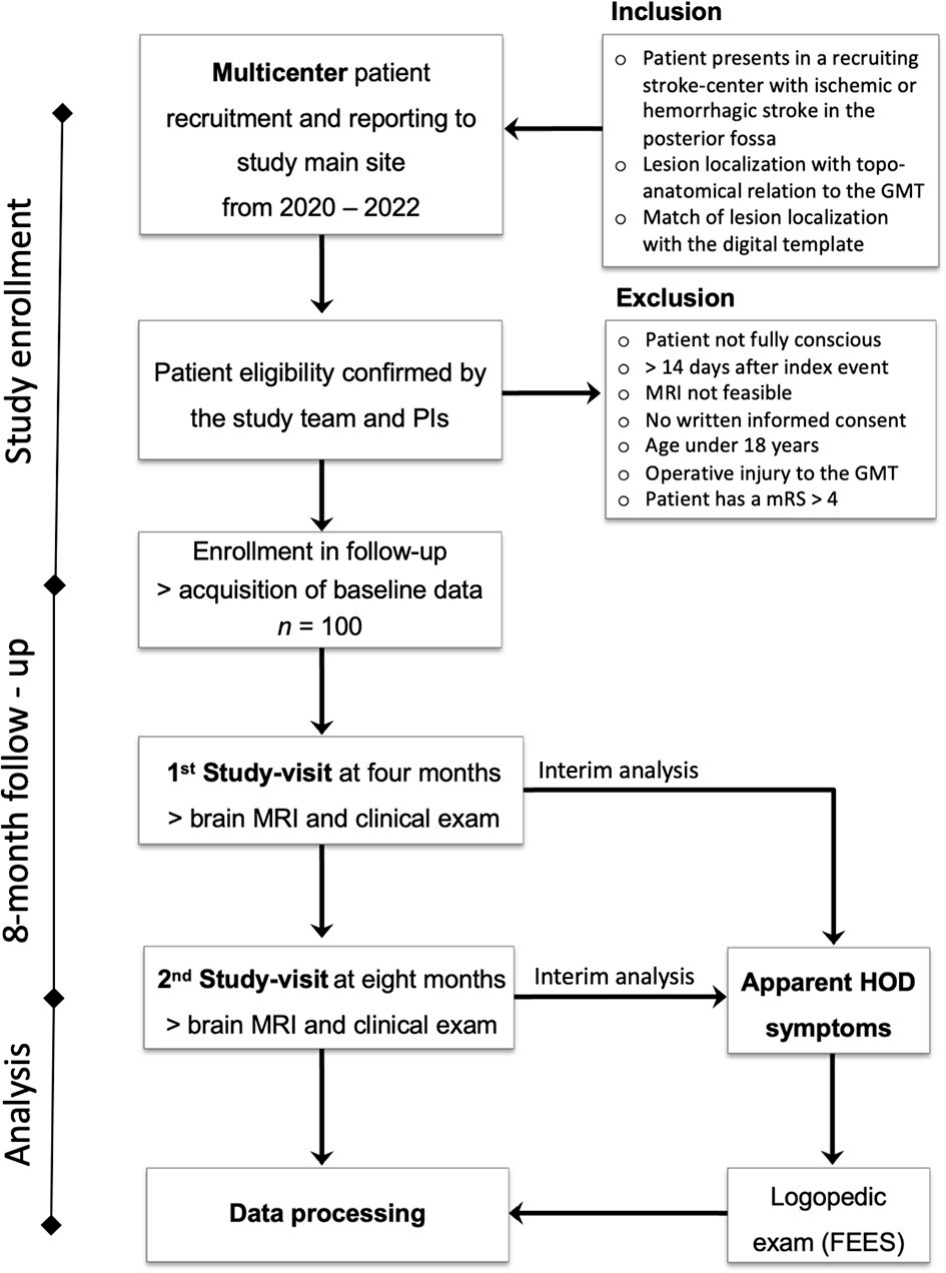
Study design chart of the HOD-IS trial.

**Figure 4:**
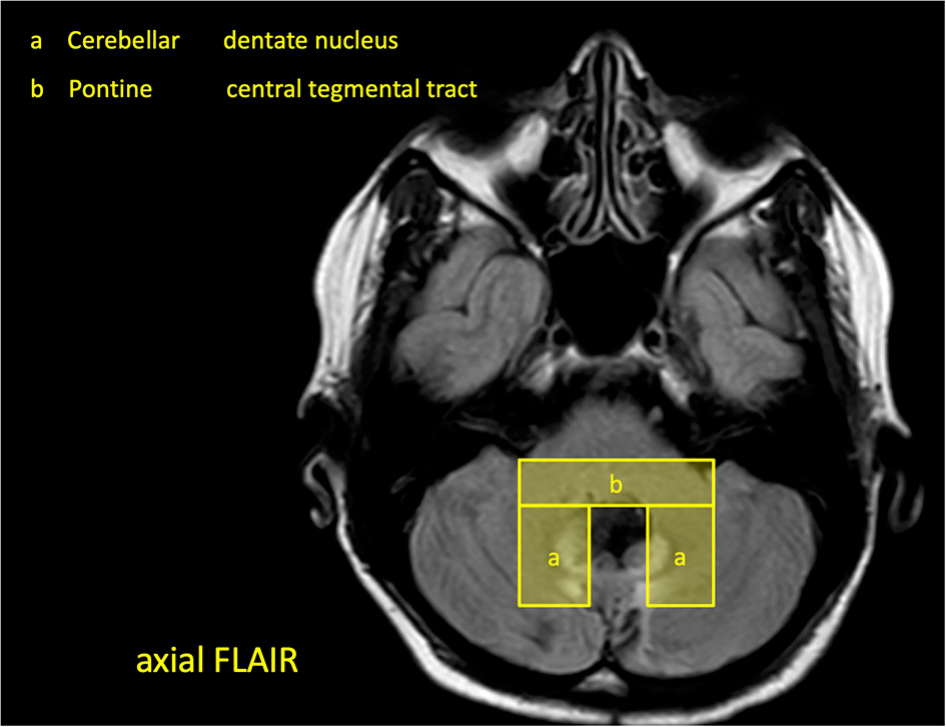
Excerpt of the study brochure, which shows an MRI template with the regions of interest.

**Figure 5:**
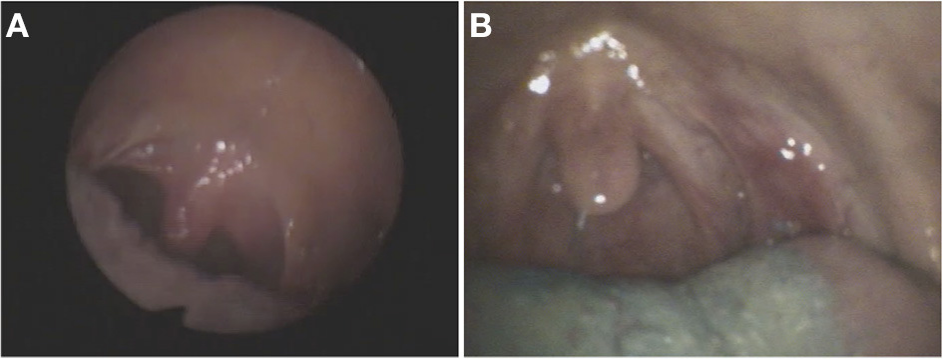
Fiberoptic endoscopic footage (FEES) of two patients **(A,B)** with HOD and dysphagia who showed involuntary movements of the soft palate and pharynx due to rhythmic contraction of the levator veli palatine, so-called palatal tremor (images used with permission from Dr. medic. Sriramya Lapa, Frankfurt).

This is a prospective multicenter study conducted in Hesse, Germany, with a 2-year-recruitment period from March 2020 to February 2022. Patients with cerebral infarctions and cerebral hemorrhages in the brainstem and cerebellum with a topo-anatomical relation to the GMT are included and followed prospectively for 8 months (Figure 3). The patients are exclusively selected based on a pre-defined digital MRI template covering the region of interest (Figure 4). During this period, two clinical neurological examinations and two follow-up MRI scans of the brain are performed at 4 and 8 months after the index event, respectively. Three major targets are defined: (1) determining the risk of HOD development for patients with ischemic or hemorrhagic lesions within the GMT, (2) defining the timeline of radiological and clinical onset of HOD, and (3) defining a real-world pattern of the clinical syndrome of HOD in clinical follow-up examinations. Three secondary targets are defined: (a) the acquisition of an additional MRI DTI protocol with fiber tractography to demonstrate the chronological decrease in fiber tract volume over time in parallel to the development of clinical symptoms and conventional radiological findings, (b) the acquisition of MRI double-echo PD-/T2-weighted images to analyze the best detectability of radiological HOD in comparison to conventional T2-weighted sequences, and (c) the assessment of PT associated dysphagia by a follow-up examination by speech therapists with Fiberoptic endoscopic evaluation of swallowing (FEES) (Figure 5) (19). The study does not include medication or the admission of computed tomography (CT) or other sources of radiation at any point.

### Sample Size

Due to the exploratory study design, an exact power calculation is not feasible. For the purpose of this study—the determination of HOD frequency after posterior fossa stroke—a maximum 10% deviation of actual disease incidence is considered within acceptable boundaries. Therefore, it is calculated that with a targeted study size of 100 study subjects, the 95% confidence limits are ≤ 10% different (absolutely) from the estimated cumulative frequency of HOD occurrence (e.g., if 30 cases are detected in *n* = 100 subjects, the 95% confidence limits are thus 0.21 and 0.40, as calculated by the Clopper-Pearson Exact method). A minimum of *n* = 100 patients is thus the targeted sample size. In addition, empirical data in the recruiting centers were assessed to approximate if sufficient patients matching inclusion criteria could be recruited. Based on yearly benchmark reports derived from the local quality assurance registry (https://www.gqhnet.de/leistungsbereiche/schlaganfall), ~6,000 ischemic and hemorrhagic stroke patients can be expected in the recruiting stroke centers per year (see below). A detailed evaluation of consecutive stroke patients from the Lausanne Stroke Registry showed that among 1,000 patients with first-ever stroke, 113 isolated brain stem infarctions, 17 isolated cerebellar infarctions, seven isolated brain stem hemorrhages, and nine isolated cerebellar hemorrhages can be expected (20). Other studies confirmed this distribution (21). Applying these numbers to the local prerequisites, an equivalent of 1,927 strokes in the brainstem and cerebellum in the 2-year study period can be estimated for the recruiting centers. Additionally, the study templates with the regions of interest were applied on 40 consecutive brainstem and cerebellar strokes in a pilot run in 2018, which showed that 35% of strokes had a general topo-anatomical relation to the GMT. Thus, in a 2-year period, 674 stroke patients with lesions affecting the region of interest can be expected in the recruiting centers. However, strict application of exclusion criteria showed that only a smaller portion of those patients could have been recruited, especially due to a lack of MR-feasibility, missing patient consent, and progressed disability due to the index stroke and advanced patient age (reflected in mRS > 4). Moreover, the SARS-Covid-19 pandemic unforeseeably complicates recruitment. Hence, the targeted number of patients to include in this study is *n* = 100, leaving a necessarily large scope for expected recruitment failures and patient exclusions. Based on these 100 patients, the proportion of patients developing HOD as well as the timeline of HOD development will be determined.

### Selection of Subjects

Patients are recruited within certified stroke centers in Hesse, Germany, which are members of the Interdisciplinary Neurovascular Network of Rhine-Main (INVN, http://invn.de/), one of 16 neurovascular networks in Germany funded by the German Stroke Society (DSG). All clinics hold a DSG-certified stroke unit and special expertise in the diagnosis and treatment of cerebrovascular diseases and neurological rehabilitation. For patient selection, a study brochure is handed out to the cooperating centers and respective radiology departments with an MRI template of imaging hallmarks of the GMT lesions (Figure 4).

### Inclusion and Exclusion Criteria

Patients are eligible for this study if they are older than 18 years and present in the recruiting centers with an intracerebral hemorrhage or cerebral infarction in the brainstem or the cerebellum not older than 14 days with topo-anatomical relation to the GMT. The region of interest is evaluated on clinical MRI or CT and predefined by the study template (Figure 4). If there is a match of lesion localization and MRI template the patient will be asked by the respective study centers to participate in the study. If there is an agreement, baseline clinical data is anonymously recorded in the study database, and the patient is contacted for follow-up (Figure 3).

Written informed consent is mandatory for recruitment. Patients are excluded from the study if explicit consent to participate in the study cannot be given due to coma or lack of legal competence. Further exclusion criteria are visibility of new operative injury on MRI (e.g., after an occipital craniotomy), contraindications to perform MR-imaging (such as pacemakers, ferromagnetic materials in the body, and claustrophobia), age under 18 years, and a modified Rankin Scale (mRS) of more than four points, which precludes transportation.

### Study Outcomes

All patients are asked to undergo follow-up MR-imaging done at the Brain Imaging Center (BIC) of the Goethe University Frankfurt. Furthermore, at the time of study visits, a clinical neurological examination is performed by a study physician following a study exam catalog. The symptoms of a rhythmic PT, pendular nystagmus, and Holmes tremor of the upper limbs are specifically evaluated by a physician with experience in the field. A complete neurological exam is performed, including gait testing. The individual disability outcome is measured by the modified Rankin Scale (mRS) during each study visit. If PT is encountered, the patient is offered an evaluation of dysphagia by a speech-language pathologist, including optional FEES.

MRI examinations are performed on a 3T whole-body MRI scanner (MAGNETOM Prisma, Siemens Healthineers, Erlangen, Germany) using a body transmit and a 20-channel phased-array head/neck receive coil (Siemens Healthineers, Erlangen, Germany). As an anatomical reference, a T1-weighted data set with whole-brain coverage and an isotropic resolution of 1 mm are acquired using a 3D magnetization-prepared rapid-gradient-echo imaging (MP-RAGE) sequence (22). PD-weighted images, acquired with a long repetition time (TR) and a relatively short echo time (TE), are compared to both T2-weighted and fluid-attenuated inversion recovery (FLAIR) images. For best detection sensitivity of radiological HOD, an infratentorial axial T2-weighted sequence of the brainstem is included as a gold standard with the following parameters: matrix size 448 × 358, FOV 240 × 240 mm^2^, TE 116 ms, TR 4,400 ms, refocusing angle 130°, 20 axial slices, slice thickness 2 mm, and slice gap 0.2 mm. PD- and T2-weighted images are recorded simultaneously via a double-echo turbo spin echo sequence (TE 12 and 96 ms) with the following parameters: matrix size 384 × 384, FOV 240 × 240 mm^2^, TR 4,500 ms, refocusing angle 130°, 25 axial slices, slice thickness 2 mm, and slice gap 0.5 mm. In correspondence with the local clinical routine protocol, FLAIR images covering the entire brain are acquired with a matrix size of 320 × 224, FOV 220 × 193 mm^2^, TR 8,500 ms, TE 81 ms, TI (inversion time) 2,440 ms, refocusing angle 150°, 30 axial slices, slice thickness 4 mm, and slice gap 0.4 mm.

**Figure 6:**
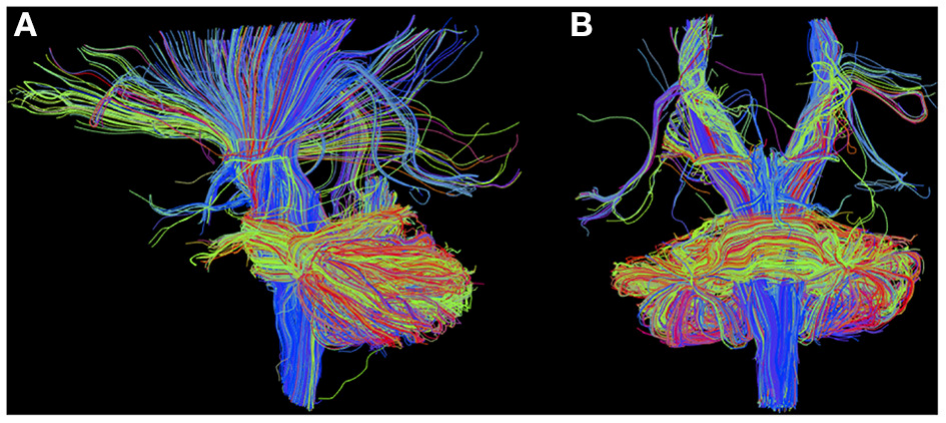
Diffusion MRI with region-of-interest-based deterministic tractography using TrackVis in sagittal **(A)** and coronal **(B)** view, rendered as described with MR data from the protocol pilot run (3T MAGNETOM Prisma, Brain Imaging Center Frankfurt) obtained from a healthy 32-year-old male test subject.

The DTI data are acquired using a novel sequence dubbed HASE-EPI sequence, as described in the literature (23). In comparison to commonly used single- and twice-refocused spin-echo EPI sequences, the HASE-EPI sequence allows for a slightly improved signal-to-noise ratio (SNR) due to the use of diffusion model independent of spatial resolution (thus shortened TE, especially for large matrix-size). The increased SNR improves the quality and quantity of the reconstructed fiber tracts. All DTI data are acquired with an isotropic resolution of 2 mm using the following parameters: FOV 256 × 256 mm^2^, matrix-size 128 × 128, 72 interleaved axial slices, slice thickness 2 mm, no inter-slice gap, TR 7,200 ms, TE 60 ms, echo-spacing 0.6 ms, bandwidth 1,953 Hz/pixel, partial Fourier encoding 6/8, parallel imaging (GRAPPA) with acceleration factor 2, 60 different diffusion weighting gradient (DWG) directions (*b*-value 1,000 s/mm^2^), and five pairs of reference (b0) volumes. The choice of directions is based on the full-sphere sampling of symmetrically distributed DWG directions. All imaging sequences used in this study are tested in pilot runs on healthy subjects prior to the start of the study (Figure 6).

FEES are performed with an Olympus ENF-P4 laryngoscope attached to a camera (rpCam62, S/N) and a color monitor (17′ -TFT-EIZO, 1,500: 1). All examinations are videotaped. FEES procedures are performed by a neurologist and a speech-language pathologist, having several years of experience with the diagnostic tool. A standardized FEES protocol will be followed strictly. All swallowing trials are rated by an experienced speech pathologist according to the Penetration Aspiration Scale.

### Data Analysis

Descriptive statistics are used to present baseline characteristics. The 95% Confidence Intervals are calculated to verify the accuracy of our primary endpoint, the frequency of HOD occurrence in patients after lesions to the GMT. Differences between groups are tested by the independent-samples *t*-test for parametric data, Pearson's Chi-square test for categorical data, and the Mann–Whitney *U*-test for non-parametric data. Other statistical tests may be used where appropriate. For all analyses, the level of significance is set at *p* < 0.05. Diffusion MRI data analysis is outlined in detail in the Supplementary Material.

### Primary Endpoint

The primary endpoint of the study is the presence of radiological HOD on T2-weighted images at the time of follow-up 8 months after the index event. The presence of radiological HOD is reviewed and validated by two experts in the field, including at least one specialist in radiology with additional neuroradiology specialty, on the basis of the brain MRI with T2-weighted sequences. The radiological diagnosis of HOD is established in consensus if a new-appearing, circumscribed hyperintensity of the ION is present on T2-weighted or FLAIR imaging, optionally accompanied by hypertrophy of the ION (4, 8, 9).

### Secondary Endpoints

Secondary endpoint values are the radiological incidence of HOD at 4 months after the index event, recorded as the main target value. Further secondary endpoints are the presence of HOD-specific symptoms in the clinical exam at follow-up and the impact of HOD development on disability outcome (mRS). The PD-weighted images of all patients will be reviewed by three blinded raters with a specialization in neuroradiology and rated for their quality of HOD identification in comparison to simultaneously acquired T2-weighted images. The DTI images will be analyzed for each individual patient with respect to lesion location within the fiber tracts of the GMT, thinning of fiber tract volume, and quantitative imaging parameters such as mean diffusivity and fractional anisotropy and will be reviewed.

## Discussion

This is the first prospective study aimed to determine the incidence of HOD following ischemic and hemorrhagic stroke with injury inflicted to the GMT. Furthermore, clinical and pathophysiological aspects of HOD will be assessed as secondary targets.

The development and clinical application of a region-of-interest template and identification of lesions in the GMT more prone to cause HOD is a key component of the study. In order to prevent a selection bias and include patients as objectively as possible, the radiological template for patient recruitment is designed to be generous in size around key structures of the GMT. Therefore, also patients with partial or minor affection of GMT structures can be included in the study, allowing for a more differentiated analysis.

In this study, brain MRI is performed at two fixed time points after the index event, which allows us to determine the disease incidence and duration until HOD visibility. Due to the nature of the study, the timestamps of study visits are set arbitrarily, and no statement is possible to distinguish HOD occurrence before or in between study visits. However, 4 and 8 months were chosen based on the expected time course of HOD based on a meta-analysis of literature (4). During the drafting of this exploratory study, it was hypothesized that an estimate of 20% of patients with a plausible lesion to the GMT will have developed HOD on MRI 8 months after the index event. This is, however, based solely on small, retrospective data and is open to be proven otherwise by the results (3, 10).

Moreover, the influence of the specific lesion location in the GMT on the probability of HOD development is still unknown. This study aims to investigate whether the effect of structures such as the DN, central tegmental tract, or RN is associated with an increased risk of HOD compared to other localizations. Knowledge of a specific incidence based on lesion location justifies a prospective follow-up of stroke patients at risk of developing HOD. Though no treatment or prophylaxis for HOD exists yet, in the future, prospective therapeutic measures could be explored, such as inhibiting the excitatory fiber tracts involved in HOD development by medicinal GABAergic modulation.

Similarly, the frequency and extent of characteristic symptoms in HOD patients are still unknown and can be firstly described in this study. No study in the literature has systematically assessed whether HOD frequently causes a clinical syndrome in the patient or is mostly a coincidental radiological finding without relevance in most. The information available in the literature is based on case reports of mostly symptomatic patients in whom diagnostic workup revealed underlying HOD, which is why the real number of asymptomatic HOD patients may lie considerably higher. Clinical follow-up examinations allow us to define a much clearer chronological pattern of the syndrome of HOD and facilitate the assignment of those symptoms to HOD in the future.

Information on the time-point of symptom onset can be used as a landmark for the clinical surveillance of patients at risk of developing HOD. This study can provide insight into whether the HOD is a disease affecting numerous patients after stroke or rather mostly a radiological phenomenon rarely of relevance to the individual. In addition, HOD symptoms may often falsely be attributed to the primary stroke lesion instead of a novel pathology if physicians fail to identify the two-stage dynamic in the patient's history and are not aware of HOD. PT in particular easily remains undetected in clinical routine if not checked for. Currently, it remains unclear if PT patients are likely to develop dysphagia or mostly suppress the tremor during the swallowing process and are not functionally affected. Improved understanding of dysphagia in PT patients is necessary to detect defective swallowing mechanisms very early and prevent silent aspiration. For this reason, if a PT is encountered in this study, an additional dysphagia assessment with FEES will be offered to the patient.

As a secondary study goal, quantitative imaging data acquired from the study cohort is utilized to provide insight into the complex brainstem connectivity. It can be assumed that the dentato-thalamo-cortical pathways behind HOD development are involved in other higher brain functions as well, as observed in the cerebellar mutism syndrome after posterior fossa surgery (3). Prior studies already applied structural and diffusion MRI data on the ION complex, and successfully tracked connections of the ventrolateral (parvocellular) subregions of the RN to the ION (24). The DTI fiber tractography is thus promising to illustrate the chronological course of fiber tract degeneration in HOD and associate the anatomical lesions from routine T2-weighted MR images to the respective fiber tract injury. Hereby, lesion areas most vulnerable for HOD development can be identified and a comparison of fiber tract volumes between symptomatic and asymptomatic HOD could be undertaken.

The HOD is not a common radiological diagnosis, and changes in conventional T2-weighted images may often be too subtle for a confident diagnosis. This study aims to provide the clinical radiologist with a more sensitive and specific sequence to confirm the suspicion of HOD: double-echo PD-weighted images with long TR are available on most clinical MR-scanners and are hypothesized to generate more contrast in the brainstem, allowing for a more certain and hereby more common diagnosis.

### Anticipated Outcomes and Limitations

This is an exploratory study design offering first-ever prospective data on epidemiological and pathophysiological aspects of HOD, which is why the outcomes cannot be anticipated. Possibly, the targeted sample of 100 patients will be too low to allow for an exact calculation of disease incidence but will provide an estimation of the magnitude. Furthermore, even though a digital template of lesion locations has been created, the expected patient cohort will likely be inhomogeneous. The distribution between ischemic infarction and hemorrhages and between cerebellar, pontine, and mesencephalic pathologies is not randomized nor matched. Thus, the findings cannot be generalized and will have to be asserted to specific lesion locations and constellations. Noteworthy, patients with severe disability, unconsciousness, and extensive lesion volume are excluded in this study. Therefore, this study does not allow for the description of the risk of HOD development in this collective of severely disabled patients, which must be taken into account when interpreting the results. An unforeseeable obstacle to overcome in 2020/2021 is surely the SARS-Covid-19 pandemic, which complicates patient recruitment as well as the availability of both imaging and clinical visits due to ever-changing local safety restrictions. To engage with changing lockdown restrictions, a scheduling tolerance of 4 weeks is granted for each study visit. This study does not include therapeutic interventions, and no medication is or will be administered. No X-rays are used. Hence, no radiation is applied to patients. MRI is performed without a contrast medium. Patients are thoroughly instructed about MRIs (such as the associated risks and complications like pacemakers, metal parts in the body, and claustrophobia) beforehand. Patients are thoroughly instructed about risks concerning FEES, for which written informed consent is mandatory.

## Ethics Statement

The studies involving human participants were reviewed and approved by the institutional Review Board of the Ethical Committee at the University Hospital Frankfurt as of February 10th, 2020 without further requests (project number: 19-467). The patients/participants provided their written informed consent to participate in this study.

## Author Contributions

MS-P conceived the study and gained ethics approval. MS-P, CF, and ES were involved in study development, literature research, and data analysis. MS, ES, MS-P, and EH analyzed and sorted imaging data. ES, MS-P, MS, RD, and EH were involved in image data processing and image development. MS-P wrote the first draft of the manuscript. TS, SW, RD, MS, SL, AS, HS, ES, CF, ST, and JK critically reviewed the study protocol, edited the manuscript, and approved the final version of the manuscript. All authors contributed to the article and approved the submitted version.

## Conflict of Interest

The authors declare that the research was conducted in the absence of any commercial or financial relationships that could be construed as a potential conflict of interest.
