# Supplementary material for: Multicenter Prospective Analysis of Hypertrophic Olivary Degeneration Following Infratentorial Stroke (HOD-IS): Evaluation of Disease Epidemiology, Clinical Presentation, and MR-Imaging Aspects
Source: Front Neurol. 2021 Jul 16;12:675123. doi: 10.3389/fneur.2021.675123 (PMC8322740; doi:10.3389/fneur.2021.675123)
Supplement: Supplementary file 1 [file Data_Sheet_1.pdf]

Diffusion MRI Data Analysis

Preprocessing and subsequent data analysis of DTI data will be performed using FMRIB's diffusion toolbox (FDT), which is a part of FSL, version 6.0.1 [1; 2]. Each dataset will be corrected for head motion, susceptibility, and eddy-current induced distortions. First, geometrical distortions caused by B0 field inhomogeneities will be corrected via TOPUP (2; 3) based on additional inverted phase-blip data. For this purpose, five pairs of reference volumes at b=0 are acquired with positive and negative phase encoding gradients for distortion correction. Then, non-brain tissue will be removed from the averaged undistorted reference images using the FMRIB brain extraction tool (BET). Final preprocessing steps for eddy-current correction will be performed via EDDY, as described in the literature [3; 4]. Voxel-wise diffusion tensor fitting will be performed using linear regression, as implemented in the FSL toolbox “dtifit” to compute fractional anisotropy (FA) maps and determine the direction of the principal eigenvectors. Probability distributions of diffusion parameters and fiber orientations for later probabilistic fiber tracking will be estimated in each brain voxel using Markov Chain Monte Carlo sampling, as implemented in BEDPOSTX [5; 6]. The number of crossing fiber populations in each voxel will be estimated using automatic relevance determination and will be limited to a maximum of three orientations.

After the underlying brainstem pathology has been identified by at least two of the researchers on high-resolution T1-weighted anatomical images, fiber tractography will be carried out. First, non-brain tissue will be removed from the anatomical image using BET with a fractional intensity threshold of 0.2 [7]. A global affine transform will then be calculated between the anatomical image and the brain-extracted average undistorted reference images acquired at b=0, using FLIRT with 6 degrees of freedom and mutual information cost function [8; 9]. Next, a cerebrospinal fluid (CSF) mask will be created using automatic segmentation of the structural image via FAST [10]. The resulting CSF map will be thresholded to exclude all non-brain voxels and voxels at the CSF-gray matter boundary from fiber tracking. To guide the probabilistic fiber tracking along the Guillain-Mollaret triangle, volumetric single seed masks are manually drawn on co-registered high-resolution T1-weighted anatomical images (MP-RAGE). The regions-of-interest (ROIs) for all necessary seed, target, and waypoint masks will be manually allocated by researchers with neuroradiological expertise to the dentate nuclei, the inferior olivary nuclei, around the red nuclei and central tegmental tracts. Exclusion masks of the midline may be applied to differentiate sides. The probabilistic tractography will then be performed on all datasets using PROBTRACKX [5; 6]. A total of 1000 samples (i.e., probability streamlines heading to both opposite directions) will be started from each seed voxel with a step length of 0.5 mm. The tracking will be stopped after 2000 steps or when the angle between two consecutive steps exceeds 78.5° or when the sample leaves the brain mask or enters the CSF mask. The sample counts per voxel will then be stored in the space of the anatomical image, yielding a connectivity distribution map per dataset. Degeneration of affected fiber tracts as identified during tractography is assessed by measuring mean diffusivity and fractional anisotropy as markers of microstructural white matter integrity and visualized. In addition, DTI data will be analyzed using other tractography software tools such as TrackVis [11] and MRtrix [12], to identify and mitigate artifacts (e.g., false positive fibre tracts). Preliminary results based on the deterministic tractography (acquired via TrackVis) are presented in Figure 6. Results based on the probabilistic tractography via FSL (PROBTRACKX) and MRtrix will be demonstrated in detail in the research paper showing the study results.

[1] M. Jenkinson, C.F. Beckmann, T.E.J. Behrens, M.W. Woolrich, and S.M. Smith, FSL. NeuroImage 62 (2012) 782–790.
[2] S.M. Smith, M. Jenkinson, M.W. Woolrich, C.F. Beckmann, T.E.J. Behrens, H. Johansen-Berg, P.R. Bannister, M.d. Luca, I. Drobnjak, D.E. Flitney, R.K. Niazy, J. Saunders, J. Vickers, Y. Zhang, N.d. Stefano, J.M. Brady, and P.M. Matthews, Advances in functional and structural MR image analysis and implementation as FSL. NeuroImage 23 Suppl 1 (2004) S208-19.
[3] M. Shrestha, P. Hok, U. Nöth, B. Lienerth, and R. Deichmann, Optimization of diffusion-weighted single-refocused spin-echo EPI by reducing eddy-current artifacts and shortening the echo time. Magma (New York, N.Y.) 31 (2018) 585–597.
[4] J.L.R. Andersson, and S.N. Sotiropoulos, An integrated approach to correction for off-resonance effects and subject movement in diffusion MR imaging. NeuroImage 125 (2016) 1063–1078.
[5] T.E.J. Behrens, H.J. Berg, S. Jbabdi, M.F.S. Rushworth, and M.W. Woolrich, Probabilistic diffusion tractography with multiple fibre orientations: What can we gain? NeuroImage 34 (2007) 144–155.
[6] T.E.J. Behrens, M.W. Woolrich, M. Jenkinson, H. Johansen-Berg, R.G. Nunes, S. Clare, P.M. Matthews, J.M. Brady, and S.M. Smith, Characterization and propagation of uncertainty in diffusion-weighted MR imaging. Magnetic resonance in medicine 50 (2003) 1077–1088.
[7] S.M. Smith, Fast robust automated brain extraction. Human brain mapping 17 (2002) 143–155.
[8] M. Jenkinson, Improved Optimization for the Robust and Accurate Linear Registration and Motion Correction of Brain Images. NeuroImage 17 (2002) 825–841.
[9] M. Jenkinson, and S. Smith, A global optimisation method for robust affine registration of brain images. Medical Image Analysis 5 (2001) 143–156.
[10] Y. Zhang, M. Brady, and S. Smith, Segmentation of brain MR images through a hidden Markov random field model and the expectation-maximization algorithm. IEEE transactions on medical imaging 20 (2001) 45–57.
[11] R. Wang, T. Benner, A. Sorensen, and V. Wedeen, Diffusion toolkit: a software package for diffusion imaging data processing and tractography. Proc Intl Soc Mag Reson Med. Vol. 15. (2007).
[12] J.-D. Tournier, F. Calamante, and A. Connelly, MRtrix: Diffusion tractography in crossing fiber regions. International Journal of Imaging Systems and Technology 22 (2012) 53-66.
